# Characterization of a Weak Allele of Zebrafish *cloche* Mutant

**DOI:** 10.1371/journal.pone.0027540

**Published:** 2011-11-23

**Authors:** Ning Ma, Zhibin Huang, Xiaohui Chen, Fei He, Kun Wang, Wei Liu, Linfeng Zhao, Xiangmin Xu, Wangjun Liao, Hua Ruan, Shenqiu Luo, Wenqing Zhang

**Affiliations:** 1 Key Laboratory of Zebrafish Modeling and Drug Screening for Human Diseases of Guangdong Higher Education Institutes, Department of Cell Biology, School of Basic Medical Sciences, Southern Medical University, Guangzhou, China; 2 Key Laboratory for Shock and Microcirculation Research of Guangdong, Guangzhou, China; 3 Department of Medical Genetics, School of Basic Medical Sciences, Southern Medical University, Guangzhou, China; 4 Department of Oncology, Nanfang Hospital, Southern Medical University, Guangzhou, China; 5 Key Laboratory of Freshwater Fish Reproduction and Development, Ministry of Education, State Key Laboratory Breeding Base of Eco-Enviroments and Bio-Resources of the Three Gorges Area, School of Life Science, Southwest University, Chongqing, China; Hong Kong University of Science and Technology, China

## Abstract

Hematopoiesis is a complicated and dynamic process about which the molecular mechanisms remain poorly understood. *Danio rerio* (zebrafish) is an excellent vertebrate system for studying hematopoiesis and developmental mechanisms. In the previous study, we isolated and identified a *cloche* ^172^ (*clo* ^172^) mutant, a novel allele compared to the original *cloche* (*clo*) mutant, through using complementation test and initial mapping. Here, according to whole mount *in-situ* hybridization, we report that the endothelial cells in *clo* ^172^ mutant embryos, although initially developed, failed to form the functional vascular system eventually. In addition, further characterization indicates that the *clo* ^172^ mutant exhibited weaker defects instead of completely lost in primitive erythroid cells and definitive hematopoietic cells compared with the *clo* ^s5^ mutant. In contrast, primitive myeloid cells were totally lost in *clo* ^172^ mutant. Furthermore, these reappeared definitive myeloid cells were demonstrated to initiate from the remaining hematopoietic stem cells (HSCs) in *clo* ^172^ mutant, confirmed by the dramatic decrease of *lyc* in *clo* ^172^ *runx1^w84x^* double mutant. Collectively, the *clo* ^172^ mutant is a weak allele compared to the *clo* ^s5^ mutant, therefore providing a model for studying the early development of hematopoietic and vascular system, as well as an opportunity to further understand the function of the *cloche* gene.

## Introduction

Hematopoiesis is a complicated and dynamic process, including an early primitive wave and a later definitive wave, which occurs in a number of anatomic locations and produces all types of blood cells throughout the lifetime of an animal [1]–[3]. In vertebrates, hematopoiesis originates from the ventral mesoderm (VM) and it has been proposed that the hematopoietic and endothelial cells arise from a common progenitor, termed hemangioblast [4]–[6]. In mice, definitive hematopoiesis is believed to be originated from an intra-embryonic tissue known as the aorta-gonad-mesonephros with the presence of the first hematopoietic stem cell (HSC) [2], [7], [8]. The HSCs then migrate to the fetal liver, the main hematopoietic organ during fetal life, and finally home to the bone marrow, where they undergo further expansion and differentiation into mature blood cells shortly after birth [2], [8]. Despite extensive studies, the molecular mechanisms of hematopoietic development and the genetic programs governing the specification, migration and survival of HSCs in these hematopoietic compartments remain poorly understood.

*Danio rerio* (zebrafish), a freshwater tropical fish that has features suitable for *N*-ethyl-*N*-nitrosourea (ENU) mutagenesis mediated large-scale forward genetic screening, is an excellent vertebrate model for studying developmental mechanisms of the hematopoietic and cardiovascular system [9]–[12]. Moreover, the hematopoietic program is highly conserved between zebrafish and mammals [13], [14], so the study of zebrafish hematopoiesis and vascular system development would also contribute to our understanding of this process in higher organisms.

The formation of vertebrate blood vessel can be subdivided into two distinct processes, vasculogenesis and angiogenesis [15]. In vertebrates, the differentiation of the hemangioblasts from the mesoderm and their subsequent migration to form the main axial vessels, which will then undergo lumen formation and artery-vein differentiation, is named "vasculogenesis" [16]. After vasculogenesis, the following sprouting and growth of new vessels from the pre-existing vessels named angiogenesis [17]. Zebrafish vascular system originates from the formation of bi-potential cells –hemangioblast from the ventral mesoderm (VM) at 6 hpf in gastrulation stage [18]. By 12 hpf these cells migrate to lateral plate mesoderm (LPM) where they differentiate to angioblast [18]. By 16 hpf, these angioblasts in LPM converge in the midline of vascular cord located between dorsal ectoderm and notochord [18]. By 28–30 hpf, the dorsal aorta (DA) and the posterior cardinal vein (PCV) can be discerned and are fully lumenized [19].

Similarly to mammals, zebrafish hematopoiesis also consists of primitive and definitive programs, and generates differentiated cells analogous to most of the mature blood lineages found in mammals [20]. Zebrafish primitive erythropoiesis originates from the posterior lateral mesoderm (PLM) as a pair of bilateral stripes at 5-somite stage [11]. These stripes subsequently extend anteriorly and posteriorly, and converge in the midline at 20-somite stage to form the main structure of the intermediate cell mass (ICM) where the erythroid progenitors further proliferate and differentiate, enter blood circulation, and finally mature at around 5–7 days post fertilization (dpf) [21]. Zebrafish primitive myelopoiesis arises from the anterior lateral mesoderm (ALM) at 10-somite stage and produces mainly macrophages and neutrophils [22], [23]. Zebrafish definitive hematopoiesis is believed to initiate from the ventral wall of dorsal aorta (VDA), an equivalence of the mouse aorta-gonads-mesonephros (AGM), with the formation of HSCs from the hemogenic endothelial cells at 26-30 hours postfertilization (hpf) [24], [25]. By 2 dpf, these HSCs in the ventral wall of DA migrate to the posterior blood island (PBI) (also referred to as caudal hematopietic tissue, CHT) [26] located between caudal artery and caudal vein, and finally home to kidney, the adult hematopoietic organ in zebrafish, by 5 dpf [27], [28].

In the previous study [29], we have isolated a differentiated myeloid lineage marker lysozyme C (*lyC*) -deficient mutant by whole-mount *in situ* hybridization (WISH), *cloche* ^172^ (*clo* ^172^), which was further confirmed as a new *cloche* mutant allele by complementation tests and positional cloning experiments [29]. The zebrafish *cloche* (*clo*) mutant, named for its bell-shaped heart, carries a spontaneous mutation characterized by severe deficiency in endothelial and blood cells, as well as the endocardium[30]. *clo* acts upstream of the genes important for hematopoietic and vascular development in zebrafish, including stem cell leukemia hematopoietic transcription factor (*scl*), the homeobox gene (*hhex*), GATA binding protein 1 (*gata1*), fetal liver kinase 1 (*flk1*), friend leukemia integration 1 (*fli1*) and ETS1-related protein (*etsrp*) [13], [31]–[34]. In addition, the defects in *clo* during vascular development are cell-autonomous, whereas those in blood cell development are both cell- and non-cell-autonomous [35]. All of these studies indicate that *clo* affects hematopoietic and endothelial cell development at a very early stage, which may be at the level of the hemangioblast. Unfortunately, the exact gene responsible for the *clo* mutant remains unknown owing to its telomeric location on chromosome 13 [31], [32]. A recent study showed that lysocardiolipin acyltransferase (*lycat)* mRNA partially rescue the blood lineage in *clo* mutants [32]. Although *lycat* is the earliest known player in the generation of both endothelial and hematopoietic lineages [32], direct evidence suggesting that *lycat* is responsible for the *clo* hematopoietic phenotype is still lacking.

Here we report that, by further characterization of both vascular and hematopoietic development in *clo* ^172^, the *clo* ^172^ mutant is a weak allele of the *clo* mutant, showing varying degrees of developmental defects in endothelial and hematopoietic cells, especially in the myeloid lineage cells. The vascular cells in *clo* ^172^ mutant were initially developed but failed to form the final functional vessels. For hematopoietic development, the primitive erythroid cells were less affected compared to the primitive myeloid cells which were totally lost in *clo* ^172^. However, the definitive hematopoiesis including erythroid and myeloid cells reappeared to some extent in *clo* ^172^ mutant. Combined with the examination of hematopoietic stem cell marker *c-myb* expression, we speculate that definitive hematopoietic cells are derived from the remaining HSCs in *clo* ^172^ mutant. Consistent with our hypothesis, the *lyc*-positive myeloid cells were greatly reduced in 3 dpf *clo* ^172^ *runx1^w84x^* double mutant compared with that in the *clo* ^172^ mutant, confirming the definitive origin of those hematopoietic cells. In summary, the *clo* ^172^ mutant, carrying a novel allele compared to the *clo* ^s5^ mutant, presents a weak endothelial and hematopoietic phenotype. The *clo* ^172^ mutant not only provides a model for studying early development of primitive and definitive hematopoietic cells, but also an opportunity to further understand the function of the *clo* gene.

## Results

### The vascular development in *clo* ^172^ mutant embryos is partially defective

**Figure 1:**
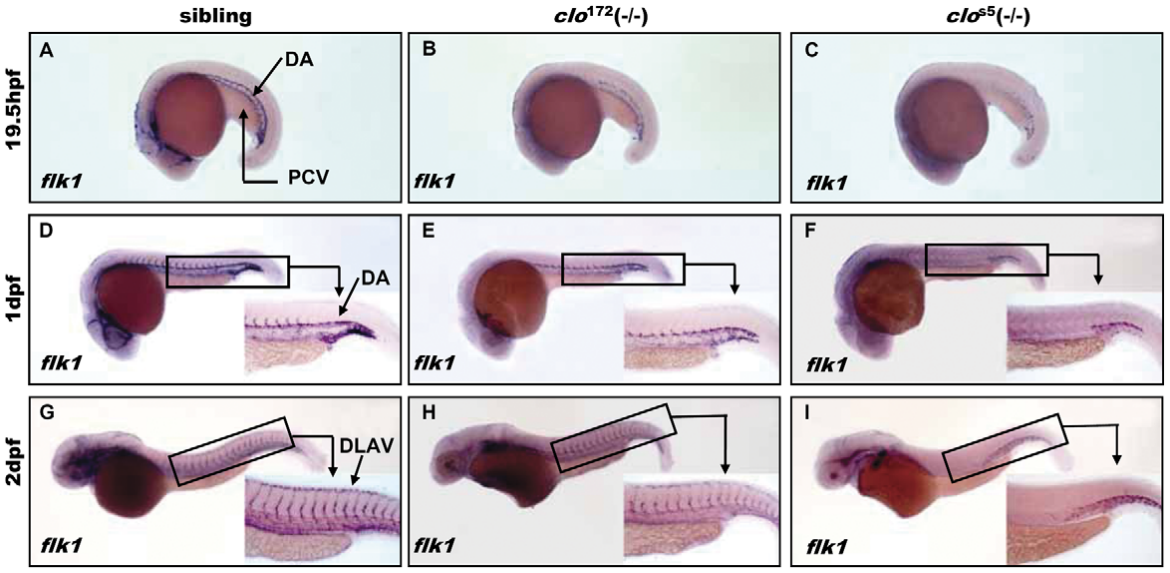
Expression of *flk1* in *clo* ^172^ and *clo* ^s5^ mutants. (**A**–**I**) Whole-mount in situ hybridization of *flk1* expression at 19.5 hpf in sibling (**A**), *clo* ^172^ mutant (**B**) and *clo* ^s5^ mutant (**C**) embryos, 1 dpf stage in sibling (**D**), *clo* ^172^ mutant (**E**) and *clo* ^s5^ mutant (**F**) embryos, and 2 dpf stage in sibling (**G**), *clo* ^172^ mutant (**H**) and *clo* ^s5^ mutant (**I**) embryos. Embryos are shown with anterior to the left and dorsal up. Inserts are high magnification (20×) of the corresponding boxed regions. DA: dorsa aorta; PCV: posterior cardinal vein; DLAV: dorsal longitudinal anastomotic vessels.

The zebrafish *clo* mutant is notable as its defects in both endothelial and blood cells at a very early stage. Therefore, we firstly explored the development of vascular system in *clo* ^172^. *Flk1,* a member of the vascular endothelial growth factor (VEGF), was utilized to perform the whole mount in-situ hybridization to characterize the development of endothelial cells and their progenitors in *clo* ^172^ mutant. The *flk1^+^* cells were found in the trunk region in *clo* ^172^ up to 19.5 hpf, which called the DA and PCV later stage, although its expression level was lower than that in siblings (**Figure 1A, B**). In 1 dpf wild type embryos, the endothelial cells were present in the head, DA, PCV and intersegmental vessels (ISV) (**Figure 1D**). In *clo* ^172^ mutant embryos, the anterior of head, DA and PCV were clearly expressing *flk1* but broken in line (**Figure 1E**), which indicated that endothelial cells were formed *in situ* but unable to connect into a tube. In 2 dpf wild type embryos, the dorsal longitudinal anastomotic vessels (DLAV) also expressed *flk1* **(** **Figure 1G**). The *flk1^+^* cells in 2 dpf *clo* ^172^ mutant embryos were faintly stained in ISV and DLAV (**Figure 1H**), which suggested that vascular system development was affected in the *clo* ^172^ mutant embryos. In *clo* ^s5^ mutant embryos, however, the expression of *flk1* was completely absent except in lower trunk and tail region (**Figure 1C, F, I**). The temporal and spatial expression pattern of *flk1* in *clo* ^172^ mutant embryos suggests that vasculogenesis, the de novo formation of vessel, is initiated but the subsequent connection is blocked, which may be related to the lumen formation and later remodeling process.

### *clo* ^172^ mutant shows severe defects in primitive myelopoiesis but partial defects in erythopoiesis

**Figure 2:**
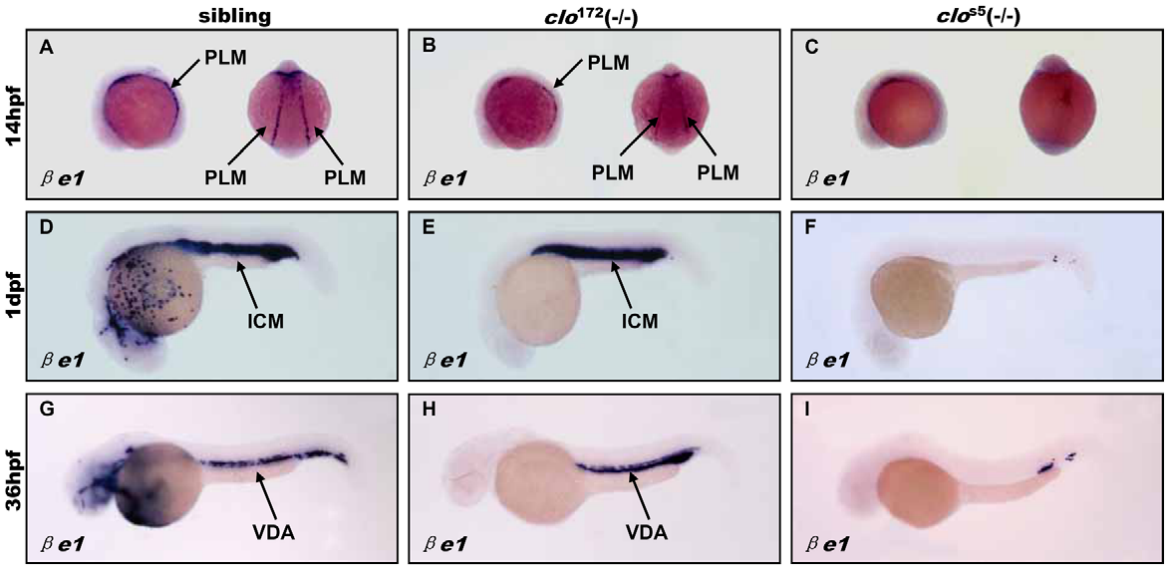
Dynamic *βe1* expression during primitive hematopoiesis in *clo* ^172^ and *clo* ^s5^ mutant embryos. (**A**–**I**) Whole-mount in situ hybridization of *βe1* expression in sibling embryos at 14 hpf (**A**)**,** 1 dpf (**D**) and 36 hpf (**G**)**,** and *clo* ^172^ mutant embryos at 14 hpf (**B**)**,** 1 dpf (**E**) and 36 hpf (**H**), and *clo* ^s5^ mutant embryos at 14 hpf (**C**)**,** 1 dpf (**F**) and 36 hpf (**I**). Embryos are shown with anterior to the left and dorsal up. PLM: posterior lateral mesoderm; ALM: anterior lateral mesoderm; ICM: intermediate cell mass; VDA: ventral wall of dorsa aorta.

To uncover the difference of hematopoietic process between *clo* ^s5^ and *clo* ^172^ mutant, we first characterized the development of primitive blood cells in *clo* ^172^ mutant at different developmental stages. The temporal and spatial expression of the erythroid lineage marker β*e1* was observed in the posterior lateral mesoderm (PLM) as two stripes at the 14 hpf stage (**Figure 2A**) and then merged to form the intermediate cell mass (ICM) at 1 dpf (**Figure 2D**), as well as in VDA region at 36 hpf (**Figure 2G**). Expression of β*e1* in *clo* ^172^ mutant embryos was partially decreased at corresponding stages (**Figure 2B, E, H**) but significantly lost in *clo* ^s5^ mutant embryos (**Figure 2C, F, I**). The different β*e1* expression between the mutants indicates that primitive erythropoiesis in *clo* ^172^ mutant is partially defective but not completely disrupted as that in *clo* ^s5^ mutant.

**Figure 3:**
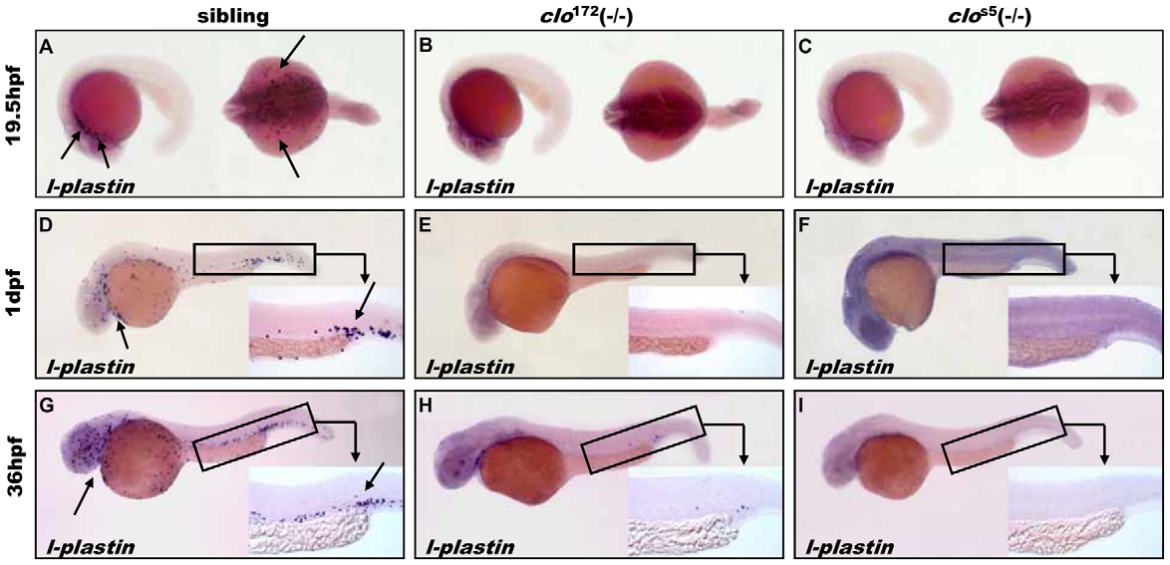
Primitive Myelopoiesis in *clo* ^172^ and *clo* ^s5^ mutant embryos. (**A–I**) Myeloid lineage marker *l-plastin* expression at 19.5 hpf(**A**: the left arrow show anterior cephalic mesoderm, **B–C**), 1 dpf stage (**D**: the left arrow show anterior cephalic mesoderm, **E–F**), and 36 hpf stage (**G**: the left arrow show anterior cephalic mesoderm **H–I**) in sibling, *clo* ^172^ and *clo* ^s5^ mutant embryos. Embryos are shown with anterior to the left and dorsal up. Inserts are high magnification (20×) of the corresponding boxed region (the right arrow show tail region).

The differentiated myeloid lineage markers *l-plastin* (**Figure 3**) and *lyC* (**Figure S2**) were utilized to detect the primitive myelopoiesis in *clo* ^172^ mutant embryos [36], [37]. In the sibling embryos, up to 19.5 hpf, the primitive myeloid cells reside in the anterior cephalic mesoderm (**Figure 3A**, Figure **S2A**). As embryos develop, myeloid cells migrate onto yolk sac syncitial layer and colonize the posterior part of tail at 1 dpf (**Figure 3D**, Figure **S2 D**), and further appear in VDA region at 36 hpf (**Figure 3G**). Largely in accordance with *clo* ^s5^ mutants (**Figure 3C, F, I**, Figure **S2 C, F**), the *l-plastin* ^+^ and *lyC* ^+^ myeloid cells were undetectable in *clo* ^172^ mutants at 19.5 hpf and 1 dpf (**Figure 3B, E**, Figure **S2 B, E**) compared to that in the same stages of siblings, whereas rare cells were observed in the tail region at 36 hpf *clo* ^172^ mutants (**Figure 3H**).

Collectively, the development of erythroid cells was not so severely affected as that of myeloid cells in the *clo* ^172^ mutant embryos during primitive hematopoiesis, in contrast to the seriously defective development of both lineages in the *clo* ^s5^ mutant.

### Myeloid lineage is partially recovered in *clo* ^172^ mutants during Definitive hematopoiesis

**Figure 4:**
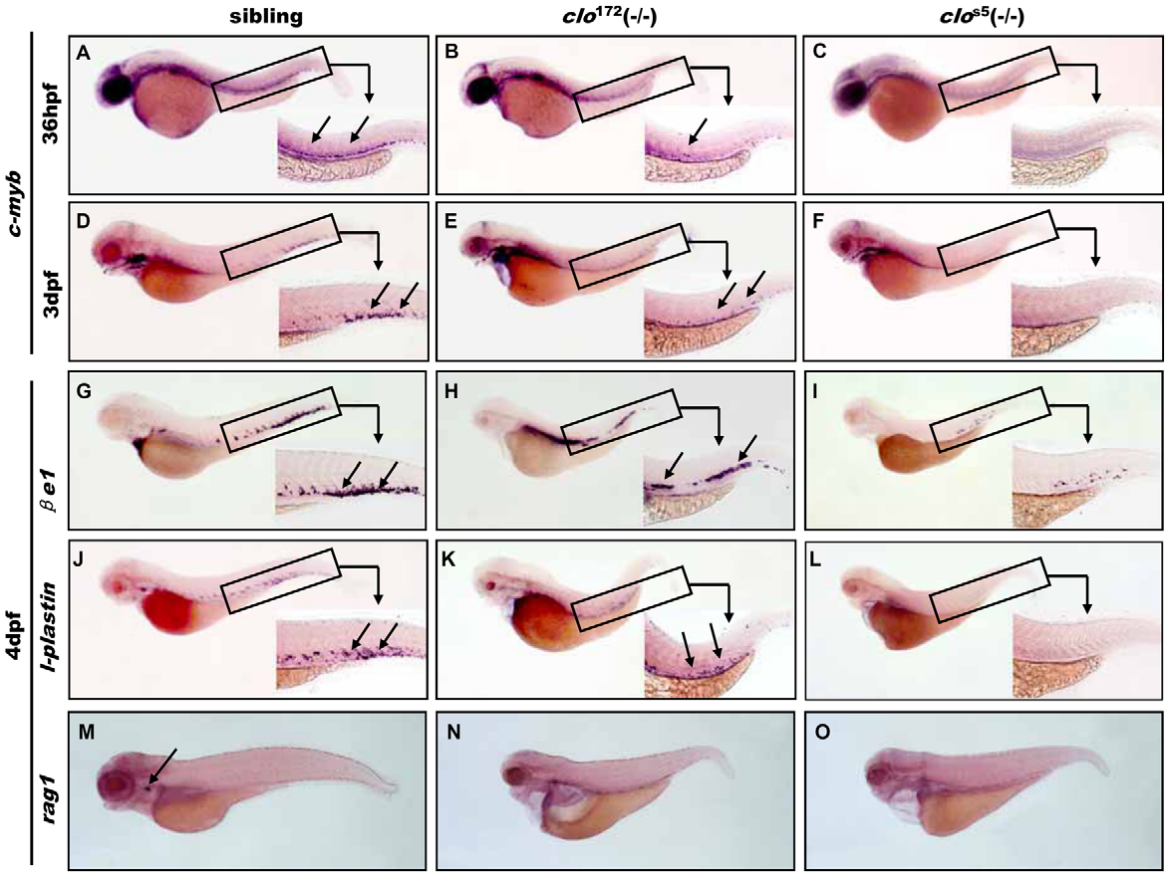
Definitive hematopoiesis in *clo* ^172^ and *clo* ^s5^ mutant embryos. (**A–C**) stem cell marker *c-myb* expression at 36 hpf and 3 dpf stage in wild type (A: arrow show VDA region; D: arrow show PBI region), *clo* ^172^ (**B, E**) and *clo* ^s5^ mutant (**C, F**) embryos.(**G–I**)**.** WISH of *βe1* expression at 4 dpf stage in sibling **(G**: arrow show PBI region**)**, *clo* ^172^ (**H**) and *clo* mutant (**I**) embryos. (**J–L**) WISH to detect *l-plastin* expression at 4 dpf stage in sibling (**J**: arrow show PBI region), *clo* ^172^ (**K**) and *clo* ^s5^ mutant (**L**) embryos. (**M–O**)WISH of T lymophcyte marker *rag1* expression at 4 dpf stage in sibling (**M**: arrow show thymus), *clo* ^172^ (**N**) and *clo* ^s5^ mutant (**O**) embryos. Embryos are shown with anterior to the left and dorsal up. Inserts are high magnification (20×) of the corresponding boxed regions. VDA: ventral wall of dorsa aorta; PBI: posterior blood island.

Our previous study [29] has showed that the red blood cells were present in VDA region in *clo* ^172^ mutant at 2 dpf (Figure **S1**), we therefore performed the WISH experiment to further examine the development of definitive hematopoiesis in *clo* ^172^ mutant. Since all the hematopoietic cells are arisen from a common ancestor known as hematopoietic stem cells (HSCs), we firstly investigated the development of HSCs in *clo* ^172^ mutant. In zebrafish definitive hematopoiesis, *c-myb* was characterized as the stem cell marker with its presence in the VDA at around 36 hpf[24], [38]. In sibling embryos, *c-myb* ^+^ cells are arranged in a line along the VDA at 36 hpf (**Figure 4A**) and assembled in tail region named as PBI at 3 dpf (**Figure4 D**). WISH indicated that the expression of HSC marker *c-myb* decreased greatly at 36 hpf and slightly at 3 dpf stage in *clo* ^172^ mutant embryos(**Figure 4B, E**). In contrast, the expression of *cmyb* was completely lost in *clo* ^s5^ mutant embryos (**Figure 4C, F**). These data indicated that HSCs are present but reduced in *clo* ^172^ mutant when compared to wild type embryos. Therefore, we speculate that HSCs were partially formed in *clo* ^172^ mutant embryos.

To examine our speculation, we further characterized *clo* ^172^ mutants to determine the development of definitive hematopoietic cells that derived from the HSCs. As show in figure 4 and figure S3, in wild type embryos, the expression of β*e1^+^* (red blood cell marker), *l-plastin* ^+^ and *lyc* ^+^ cells (myeloid cell marker) were normally localized in PBI region at 3 and 4 dpf stage (**Figure 4G, J**, **Figure S3 A, D**). No obvious differences in β*e1* expression were observed between *clo* ^172^ mutants and siblings, except that red blood cells located in the VDA region (**Figure 4H**) at 4 dpf, which may be due to the lack of blood circulation in the *clo* ^172^ mutants. In contrast, β*e1^+^* cells were nearly undetectable in the *clo* ^s5^ mutant (**Figure 4I**). On the other hand, the examination of the expression of myeloid lineage markers *l-plastin and lyc* indicated that myeloid cells were decreased but ectopically expressed in a gradient manner in VDA in *clo* ^172^ mutants at 3 dpf and 4 dpf (**Figure 4K**, **Figure S3 B, E**) compared with wild-type siblings, whereas they were also undetected in the *clo* ^s5^ mutant at 3 dpf (**Figure 4L**, **Figure S3 C, F**). Furthermore, investigation of the T-lymphocyte developmental marker *rag1* revealed that it was totally lost in thymus in both *clo* ^172^ and *clo* ^s5^ mutants at 4 and 5 dpf (**Figure 4N, O**, **Figure S3 H, I**). These data reveal that definitive erythropoiesis and myelopoiesis, but not lymphocyte, are more or less defective in the *clo* ^172^ mutant embryos. Additionally, the development of the definitive myeloid lineage was obviously different from its primitive counterpart.

Taken together, the results suggest that hematopoietic cells partially develop in the *clo* ^172^ mutant during definitive hematopoiesis, which shows a weak phenotype compared to the *clo* ^s5^ mutant.

### Definitive myeloid lineage cells in *clo* ^172^ mutant originated from the remaining HSCs population

**Figure 5:**
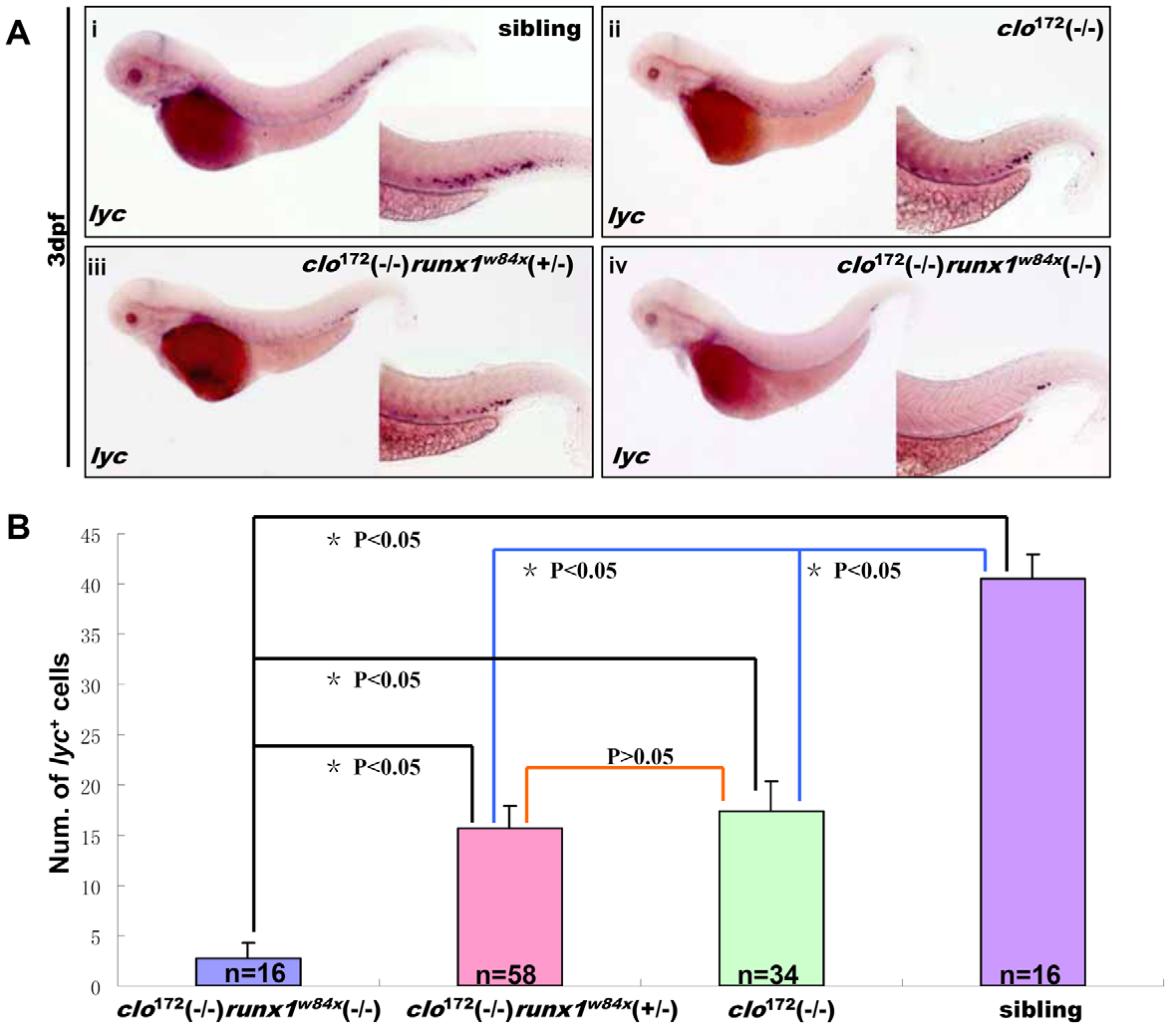
Analysis of the origin of definitive myeloid cells in *clo* ^172^ mutant. **(A, B)** *lyc* expression pattern at 3 dpf *runx1*/*clo* ^172^ double mutant (A). WISH of *lyc* expression in siblings (A–i), *clo* ^172^ mutant (A–ii), *clo* ^172^ (-/-) *runx1* (+/-) *^w84x^* (A-iii), double homozygous mutant (A–iv). Histogram of *lyc* ^+^ cells number (means) in siblings(B, purple column), *clo* ^172^ mutant (B, green column), *clo* ^172^ (-/-) *runx1* (+/-)*^w84x^* (B, pink column) and double homozygous mutant (B, blue column). There are significant differences between double mutant and other groups (*p<0.05), but no difference between *clo* ^172^ mutant and *clo* ^172^ (-/-) *runx1* (+/-) *^w84x^* group (p>0.05). The corresponding case numbers were shown by n in column.

Since the HSCs were partially formed in the *clo* ^172^ mutant, we therefore continued to exam whether the presented definitive myeloid cells were differentiated from the remaining HSCs. Thus, we introduced *runx1*mutation, the well-known gene required for the definitive hematopoiesis in zebrafish[39], [40], into the *clo* ^172^ mutant background by crossing *clo* ^172^ (+/-) mutant with *runx1^w84x^* mutant[41], and then examined the development of definitive myeloid cells in their offspring. The embryos showing *clo* ^172^ mutant morphology change (no circulation, edema of heart) were firstly selected and fixed at 3 dpf, and applied to WISH to detect *lyc*. The number of *lyc* ^+^ cells was counted and each fish was genotyped for *runx1* mutation. The results showed that *lyc* ^+^ cells were dramatically decreased in PBI region of *clo* ^172^(-/-)*runx1^ w84x^* (-/-)embryos compared to that in *clo* ^172^(-/-) embryos at 3 dpf stage (**Figure 5A-ii, iii, iv**) (**Figure 5A-i**). Remarkably, the statistic analysis by ANOVA statistic method showed that there were significant differences (p<0.05)in *lyc* ^+^ cell number among *clo* ^172^(-/-)*runx1^ w84x^* (-/-)group (case number = 16), *clo* ^172^(-/-)*runx1^ w84x^* (+/-)(case number = 58) group, *clo* ^172^(-/-) group (case number = 34), and siblings (case number = 16) (**Figure 5B**) and there was no significant differences (p>0.05)between *clo* ^172^ (-/-) *runx1^ w84x^* (+/-)group and *clo* ^172^(-/-) group. Thus, our data suggest the definitive origin of the presented myeloid cells in 3 dpf *clo* ^172^ mutant, which were derived from the remaining HSCs in the *clo* ^172^ mutant through a *runx1*-dependent manner.

## Discussion

The *clo* ^172^ mutant embryos are identified by a lack of blood circulation but no obvious morphological changes (**Figure S1B**) before 30 hpf. Similar to *clo* ^s5^ mutants, *clo* ^172^ mutants gradually exhibit slight swelling of the heart and morphological changes from 2 dpf onward and thereafter (**Figure S1E, F**). Although the morphological changes are similar to those in *clo* ^s5^ mutants, the red blood cells are clearly visible and localized in the VDA region of *clo* ^172^ mutants (**Figure S1E**). These data (detailed description in Materials S1) suggested that the *clo* ^172^ mutant was a different allele compared to the *clo* ^s5^ mutant. Thus, the *clo* ^172^ mutant was used for further characterization of vasculogenesis and hematopoiesis, and the results were compared to those from *clo* ^s5^ mutants to identify differences between these two mutants.

WISH of *flk1* in *clo* ^172^ mutants revealed that vascular endothelium cells were able to form *in situ* but unable to connect into tubes. We predict that vascular system development is normally initiated but finally fails to form the functional vascular system, which may be defective in lumen formation and later remodeling process.

Unlike *clo* ^s5^ mutants, in which the development of all lineages in hematopoiesis is severely defective, the *clo* ^172^ mutant shows partial defects in both primitive and definitive erythropoiesis. By contrast, myelopoiesis is more complicated in the *clo* ^172^ mutant. An interesting issue raised by this study is that the development of primitive myeloid cells is seriously affected, whereas the definitive myelopoiesis is partially restored in the *clo* ^172^ mutant. According to the expression of HSC marker *c-myb*, we observed dramatically decreased but still retained *c-myb* ^+^ cells in the *clo* ^172^ mutant during definitive hematopoiesis. Due to the remainig HSCs present in the *clo* ^172^ mutant, we predicted that definitive erythroid and reappeared myeloid cells might originate from them. Based on Nancy A. Speck's report[39], which noted that *runx1* was required for the emergence of hematopoietic stem cells (HSCs) from hemogenic endothelium during embryogenesis, we speculated that *lyc* ^+^ cells in the 3 dpf *clo* ^172^ mutant would greatly decreased in PBI region due to the loss of *runx1* gene. To validate our speculation, we crossed the *runx1^w84x^* mutant with *clo* ^172^ mutant and examined the expression of *lyc* in their offspring. The results showed that *lyc* ^+^ myeloid cells were dramatically reduced in PBI region at 3 dpf stage in *runx1^w84x^clo* ^172^ double mutant, therefore confirming their definitive origin through the *runx1* dependent manner. In addition, it is notable that the blood cell migration is probably dependent on blood flow [42]. Hence, we speculate that visible red blood cells and reappeared myeloid cells existing in the VDA region in the *clo* ^172^ mutant might be caused by the lack of blood circulation.

Our study has provided evidence that the *clo* ^172^ mutant is a weak allele compared to the *clo* ^s5^ mutant with partially developed primitive and definitive hematopoiesis, thus providing a model for studying the early development of hematopoietic and vascular system, as well as an opportunity to further investigate the function of *clo* gene in genetic pathways.

## Materials and Methods

### Ethics statement

All experimental protocols and animals used in this research were approved by Ethical Committee of Southern medical University (LS2011–030).

### Zebrafish husbandry

Zebrafish were raised and handled as previously described [43], [44]. The *cloche* ^s5^ mutant used in this study was kindly provided by Dr. Didier Y.R. Stainier's laboratory (University of California at San Francisco). The *runx1 ^w84x^* mutant [41] was used to cross with *clo* ^172^ (+/-) to generate *clo* ^172^ (+/-) *runx1^ w84x^* (+/-).

### *In vitro* synthesis of antisense RNA probes

Antisense RNA probes were prepared by *in vitro* transcription according to a standard protocol [43]. The following probes were used in the study: digoxigenin (DIG)-labeled antisense β*e1-globin, lyC*, *l-plastin*, *rag-1*, *c-myb, runx1* and *flk1*.

### Whole-mount *in situ* hybridization

Embryos at different stages were used to examine hematopoietic-related gene expression by WISH as previously described[43]. Consistent with the Mendelian law of heredity, the ratio of mutant to total embryos was approximately 1/4 (**Table 1**).

**Table 1:** No. of embryos used in WISH.

Probe	Stage of embryos	*clo* ^172^ mutant(mutant/total)	*clo* ^s5^ mutant(mutant/total)
*flk*1	21 s	39/156	31/133
*flk*1	1 dpf	28/117	24/102
*flk*1	2 dpf	25/104	23/97
β*e1*	14 hpf	52/219	54/221
β*e1*	1 dpf	37/156	35/149
β*e1*	36 hpf	58/208	54/200
β*e1*	4 dpf	34/132	38/145
*l-plastin*	19.5 hpf	23/94	29/123
*l-plastin*	1 dpf	39/152	41/163
*l-plastin*	36 hpf	53/206	49/197
*l-plastin*	3 dpf	41/171	38/154
*l-plastin*	4 dpf	37/152	33/149
*c-myb*	36 hpf	37/152	34/147
*c-myb*	3 dpf	27/116	31/127
*Rag-1*	4 dpf	22/93	25/103
*rag1*	5 dpf	48/189	44/181
*lyC*	19.5 hpf	36/159	40/165
*lyC*	1 dpf	43/165	41/162
*lyC*	3 dpf	53/209	49/201

**M: No. of mutant embryos; T: No. of total embryos.**

### Genotyping of *runx1* gene

The *clo* ^172^ (-/-) embryos were picked out from *clo* ^172^ (+/-) *runx1^ w84x^* (+/-) offspring based on the phenotype of no circulation at 28 hpf. Then these embryos were used to check the level of *lyc* by WISH. After washing with PBST, the genomic DNA was extracted from each embryo for the PCR amplification using *runx1* primers followed: 5′-TGGTGGGCAAACTGCGCATG-3′ and 5′-TTCTTGCTGTGACACTGAGC-3′. Subsequently, the genotype of sibling versus mutants was easily distinguished by the different size of the PCR product after digestion by the Hae II enzyme.

## Supporting Information

Figure S1 **Dynamic morphological changes of** ***clo*** **^172^ and** ***clo*** **^s5^ mutant.** (A–F) Lateral view of morphological changes 30 hpf stage of sibling **(A),** *clo* ^172^ **(B),** *clo* ^s5^ mutant **(C) and** 2 dpf stage of sibling **(D)**, *clo* ^172^ **(E**: arrow show edema heart and red blood cell in VDA region**),** *clo* ^s5^ mutant **(F**: arrow show edema heart**)**.
(TIF)

Figure S2 **Expression of** ***lyc*** **during primitive hematopoiesis in** ***clo*** **^172^ and** ***clo*** **^s5^ mutant embryos. (A**–**F)** Whole-mount in situ hybridization of *lyc* expression at 19.5 hpf (**A**: arrow show anterior cephalic mesoderm, **B**–**C**), 1 dpf stage (**D**: the left arrow show anterior cephalic mesoderm, **E**–**F**) in sibling, *clo* ^172^ mutant and *clo* ^s5^ mutant embryos. Embryos are shown with anterior to the left and dorsal up. Inserts are high magnification (20×) of the corresponding boxed regions.
(TIF)

Figure S3 **Definitive myelopoiesis and** ***rag1*** **expression pattern in** ***clo*** **^172^ and** ***clo*** **^s5^ mutant embryos.** *l-plastin* and *lyc* expression at 3 dpf in sibling(A, D), *clo* ^172^ **(B, E),** *clo* ^s5^ mutant **(C, F).** ***rag1*** expression at 5 dpf in sibling**(G)**, *clo* ^172^ **(H),** *clo* ^s5^ mutant **(I).** Inserts are high magnification (20×) of the corresponding boxed regions (the right arrow show tail region).
(TIF)

Materials S1 **Supporting materials.**
(DOC)
