# Supplementary figures and images for: Characterization of a Weak Allele of Zebrafish cloche Mutant
Source: PLoS One. 2011 Nov 23;6(11):e27540. doi: 10.1371/journal.pone.0027540 (PMC3223178; doi:10.1371/journal.pone.0027540)

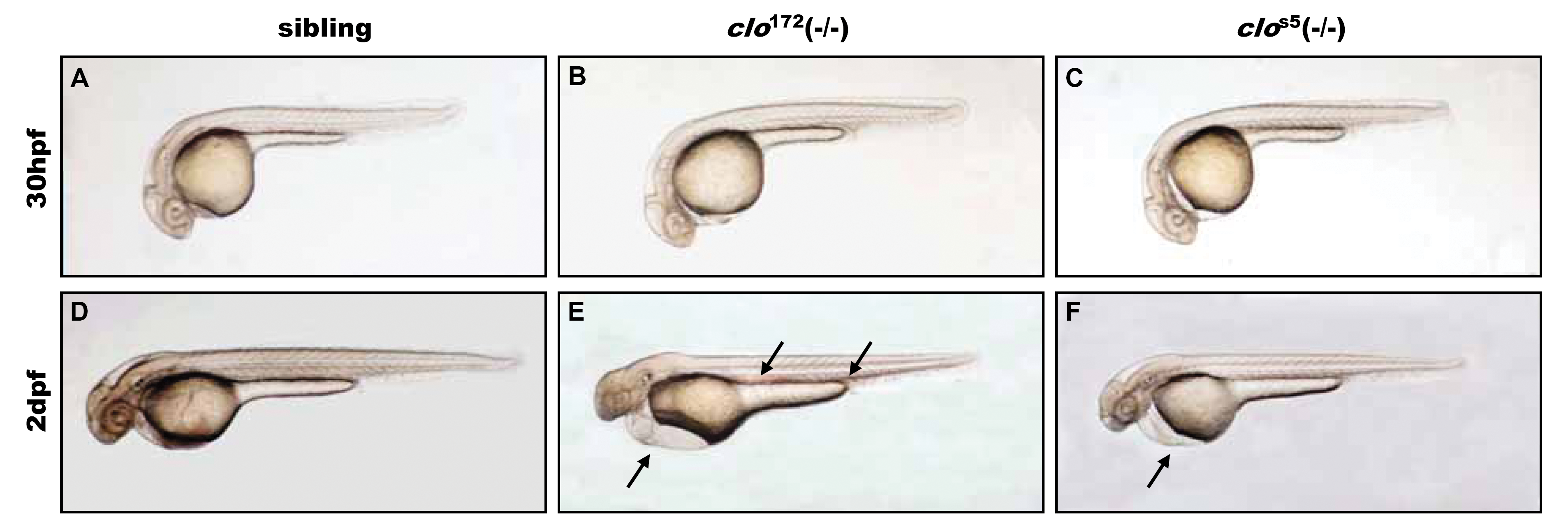

Supplement: Figure S1 — Dynamic morphological changes of clo 172 and clo s5 mutant. (A–F) Lateral view of morphological changes 30 hpf stage of sibling (A), clo 172 (B), clo s5 mutant (C) and 2 dpf stage of sibling (D), clo 172 (E: arrow show edema heart and red blood cell in VDA region), clo s5 mutant (F: arrow show edema heart). (TIF) [file pone.0027540.s001.tif]

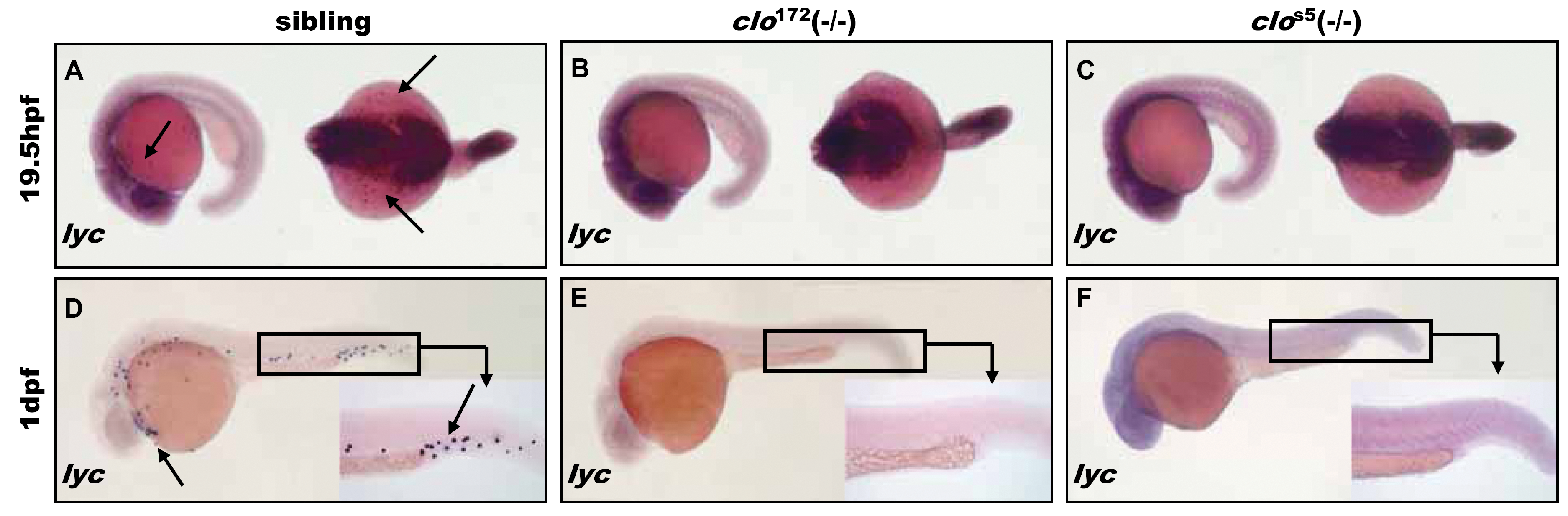

Supplement: Figure S2 — Expression of lyc during primitive hematopoiesis in clo 172 and clo s5 mutant embryos. (A–F) Whole-mount in situ hybridization of lyc expression at 19.5 hpf (A: arrow show anterior cephalic mesoderm, B–C), 1 dpf stage (D: the left arrow show anterior cephalic mesoderm, E–F) in sibling, clo 172 mutant and clo s5 mutant embryos. Embryos are shown with anterior to the left and dorsal up. Inserts are high magnification (20×) of the corresponding boxed regions. (TIF) [file pone.0027540.s002.tif]

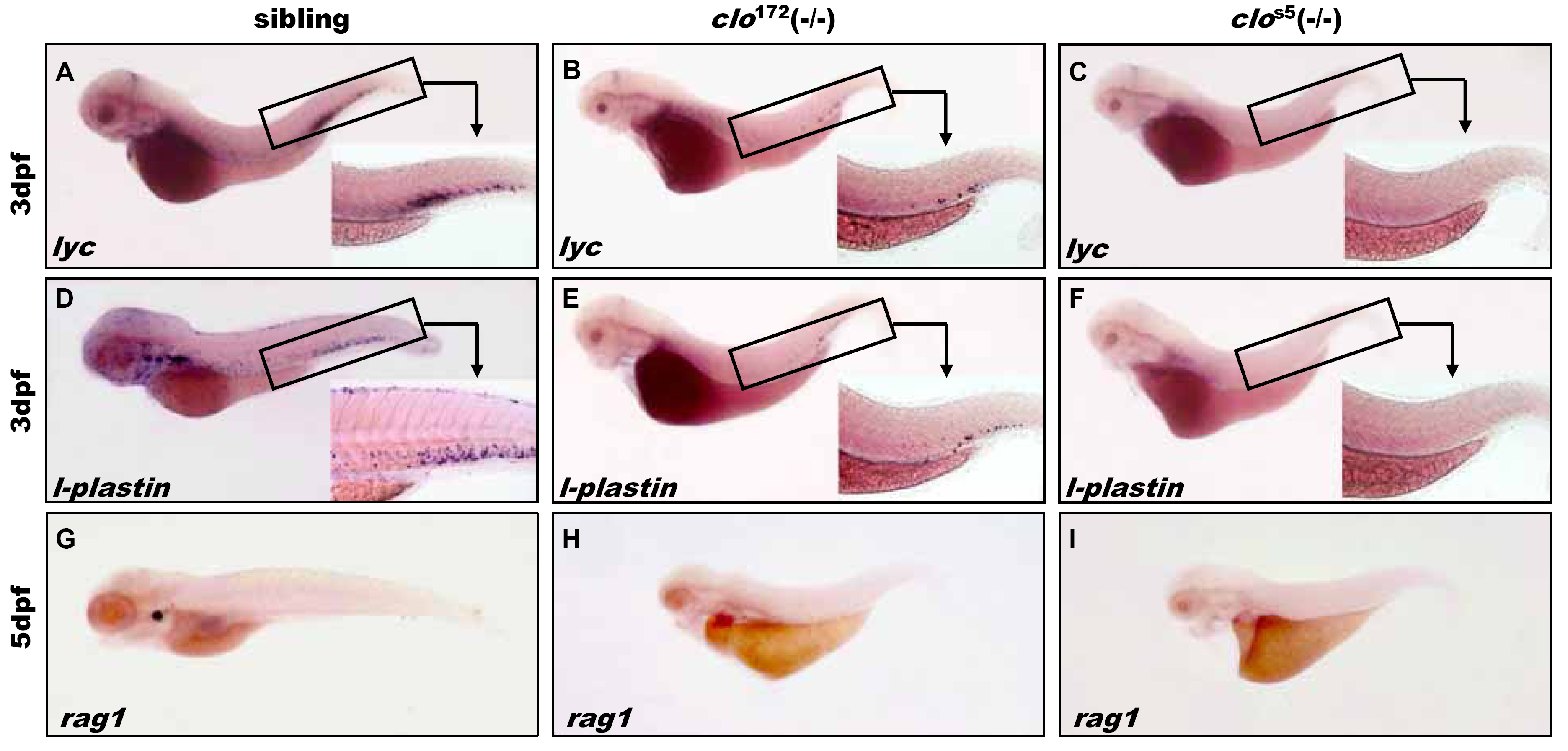

Supplement: Figure S3 — Definitive myelopoiesis and rag1 expression pattern in clo 172 and clo s5 mutant embryos. l-plastin and lyc expression at 3 dpf in sibling(A, D), clo 172 (B, E), clo s5 mutant (C, F). rag1 expression at 5 dpf in sibling(G), clo 172 (H), clo s5 mutant (I). Inserts are high magnification (20×) of the corresponding boxed regions (the right arrow show tail region). (TIF) [file pone.0027540.s003.tif]
