# Supplementary material for: Characterization of a Weak Allele of Zebrafish cloche Mutant
Source: PLoS One. 2011 Nov 23;6(11):e27540. doi: 10.1371/journal.pone.0027540 (PMC3223178; doi:10.1371/journal.pone.0027540)
Supplement: Materials S1 — Supporting materials. (DOC) [file pone.0027540.s004.doc]

**Supporting materials**

***clo*^172^ mutant shows different morphological changes compared to the *clo*^s5^ mutant**

To further comparing morphological difference between *clo*^172^ and *clo*^s5^ mutants, we observed the live embryos from each mutant in a parallel way for one week. From the beginning of circulation, both *clo*^172^(mutant/total embryos 43:167) and *clo*^s5^(mutant/total embryos 48:198) homozygotes show the similar phenotype such as defect of blood circulation (from 26-28hpf and data not shown), gradually swelling of the heart (from 30hpf) , weak heartbeat (from 2dpf onward) (**Figure S1 B, C, E, F**), and similar survival duration (approximately 6 days). However, the most significant morphological difference in between is the visible red blood cells in the VDA region of *clo*^172^ but not *clo*^s5^ mutant embryos at 2 dpf (**Figure S1 E**). These data suggest that the *clo*^172^ mutant has a different phenotype compared to the *clo*^s5^ mutant.
